# Arkansas State Medical Society

**Published:** 1882-02-20

**Authors:** 


### *ARKANSAS STATE MEDICAL SOCIETY.*

A medical friend has kindly mailed us the Minutes of the State Medical Society of Arkansas, held at Little Rock, on April 7th, 1881.

The officers elected for the year are:

Dr. R. G. Jennings, President; Dr. G. B. Malone, First Vice-President; Dr. D. C. Ewing, Second Vice-President; Dr. H. H. Turner, Third Vice-President; Dr. W. H. Heard, Fourth Vice-President; Dr. L. P. Gibson, Secretary; Dr. Ed. Meek, Assistant Secretary; Dr. A. L. Breysacher, Treasurer; Dr. Jno. Watson, Librarian.

Dr. W. M. Lawrence, the President, in his annual address, thus alludes to the late law in regard to the practice of medicine in Arkansas:—

"It is wonderfully catholic. It accepts all creeds, dogmas and articles of faith. No one is excluded on account of "race, color or previous condition of servitude." Any man or woman, over twenty-one years of age, of good moral character, who has been engaged in a so-called reputable practice for a period of five years, with the county clerk's certificate, is a doctor *de jure*, if not *de facto*, and has a right, whether qualified or not, to practice when and where, or how he or she may please. Emulation in the profession of medicine is as laudable as in any other pursuit of life; but the act suggests nothing beneficial to the old; nothing to encourage a praiseworthy ambition in the young; it gives no merit marks; on the contrary, pulls all down to the same obnoxious, common level, requiring only a probationary period of five years, without looking even to an ordinary common education. A reputable practice and the reputability of the professional services is to be determined, not by professional men, but by the several county clerks who may be, and most probably are, wholly ignorant of the knowledge and management of cases involving life, health and happiness."

The next meeting of the Society will be on Wednesday next preceding the meeting of the American Medical Association, May, 1882.

